# Serum Angiopoietin-1 and Angiopoietin-2 as Biomarkers of Pulmonary Hemodynamic Burden in COPD-Associated Pulmonary Hypertension: A Cross-Sectional Study

**DOI:** 10.3390/arm94040059

**Published:** 2026-08-11

**Authors:** Jyoti Bajpai, Akshyaya Pradhan, Mohammed Kaleem Ahmad, Surya Kant, Ajay Kumar Verma, Darshan Kumar Bajaj, Rishi Sethi, Mario Cazzola

**Affiliations:** 1 Department of Respiratory Medicine, KGMU, U.P., Lucknow 226003, India; skantpulmed@gmail.com (S.K.); bajajdarshan@gmail.com (D.K.B.); 2 Department of Cardiology, KGMU, U.P., Lucknow 226003, India; akshyaya33@gmail.com (A.P.); drrishisethi1@gmail.com (R.S.); 3 Department of Biochemistry, KGMU, U.P., Lucknow 226003, India; kaleembaksh@gmail.com; 4 Department of Respiratory Medicine, RML Institute of Medical Sciences, Lucknow 226010, India; drajay21@gmail.com; 5 Dipartimento di Medicina dei Sistemi, University of Rome Tor Vergata, 00133 Rome, Italy

**Keywords:** pulmonary hypertension, chronic obstructive pulmonary disease, angiopoietin-1, angiopoietin-2, biomarkers, echocardiography, vascular remodeling, non-invasive assessment

## Abstract

**Highlights:**

**What are the main findings?**

- Pulmonary vascular change in COPD-PH: Compared to those with normal pulmonary arterial pressure, COPD patients with pulmonary hypertension had considerably higher serum levels of angiopoietin-1 and angiopoietin-2.
- Correlation with hemodynamic burden: Increased non-invasively estimated mean pulmonary artery pressure and right atrial pressure are strongly correlated with higher levels of circulating ANGP-1 and ANGP-2.

**What are the implications of the main findings?**

- Understanding of vascular remodeling: These results emphasize the part that angiogenic growth factors play in the pathophysiology and vascular remodeling that underlie pulmonary hypertension linked to COPD.
- Non-invasive screening potential: Serum angiopoietins may be useful as accessible biomarker candidates to help with non-invasive risk assessment for pulmonary hypertension in COPD.

**Abstract:**

**Purpose:** Pulmonary hypertension (PH) is a serious complication of COPD associated with worse outcomes. Reliable circulating biomarkers of pulmonary vascular involvement are lacking. Angiopoietin-1 (ANGP-1) and angiopoietin-2 (ANGP-2) regulate endothelial homeostasis, but their relationship with echocardiographically estimated pulmonary pressure in COPD remains uncertain. **Methods:** This prospective cross-sectional study included 70 consecutively recruited adults with COPD, stratified using a study-specific echocardiographic threshold into the COPD PH group (mPAP > 20 mmHg; *n* = 46) and the COPD without PH (mPAP < 20 mmHg; *n* = 24), together with 20 additional controls. This non-invasive stratification was not considered equivalent to PH confirmed by right heart catheterization. Serum ANGP-1 and ANGP-2 were measured using ELISA. Group differences and associations with e-mPAP and right atrial pressure (RAP) were assessed. **Results:** Median ANGP-1 was higher in the elevated e-mPAP group [8780.06 (IQR 7955.29–9902.88) pg/mL] than in the lower e-mPAP group [3065.14 (1170.69–6200.41)] and controls [2997.19 (1292.13–3278.18); *p* < 0.001]. ANGP-2 showed a similar pattern [6152.92 (5380.27–8652.50), 2669.77 (1802.18–4744.00), and 2405.54 (1779.73–3751.76) pg/mL; *p* < 0.001]. Among COPD participants, ANGP-1 and ANGP-2 correlated with e-mPAP (r = 0.64 and r = 0.54) and RAP (r = 0.54 and r = 0.52; all Holm-adjusted *p* < 0.001). In multivariable models, e-mPAP and RAP were independently associated with ANGP-1, whereas RAP was independently associated with ANGP-2. **Conclusions:** Higher circulating ANGP-1 and ANGP-2 were associated with an elevated echo-estimated pulmonary-pressure phenotype in COPD. These findings are exploratory and require validation in larger cohorts using direct hemodynamic assessment.

## 1. Introduction

Chronic obstructive pulmonary disease (COPD) is the third leading cause of death worldwide. It imposes a major public health burden through progressive airflow limitation and multiple comorbidities that worsen morbidity and mortality [1,2]. Among these, pulmonary hypertension (PH) is particularly consequential, being associated with reduced exercise capacity, increased hospitalizations, and higher mortality [3].

According to the 7th World Symposium on Pulmonary Hypertension, PH associated with COPD is classified as Group 3 PH and represents a continuum of pulmonary vascular involvement [4]. The current hemodynamic definition of PH is a mean pulmonary arterial pressure (mPAP) > 20 mmHg at rest measured by right heart catheterization (RHC), with pulmonary vascular resistance and pulmonary arterial wedge pressure used for further classification [5]. Echocardiography estimates pulmonary pressure and the probability of PH, but does not establish a definitive hemodynamic diagnosis. Accordingly, the present study used echo-estimated mPAP categories as a study-specific non-invasive pulmonary-pressure phenotype.

Right heart catheterization remains the reference standard for diagnosing and classifying PH [2,5]. Its invasiveness, cost, and procedural requirements limit routine use in all patients with COPD. Transthoracic echocardiography (TTE) is therefore recommended as the initial non-invasive screening method and provides estimates of pulmonary pressure and right-heart structure and function [5,6]. However, TTE is less precise than RHC and cannot directly characterize pulmonary vascular resistance or confirm PH, supporting investigation of complementary circulating biomarkers [7].

Validated PH biomarkers include asymmetric dimethylarginine, endothelin-1, von Willebrand factor, and natriuretic peptides (brain natriuretic peptide [BNP] and N-terminal pro–BNP [NT-pro-BNP]) [8,9,10,11,12,13,14]. BNP and NT-pro-BNP correlate with right ventricular dysfunction and prognosis but reflect myocardial strain rather than pulmonary vascular remodeling per se [13,14,15]. Consequently, there is growing interest in endothelium-derived mediators that more directly reflect vascular remodeling.

Angiopoietins (ANGP-1 and ANGP-2) are endothelial-derived glycoproteins that regulate vascular stability and permeability via the Tie2 receptor [16,17,18]. ANGP-1 maintains endothelial integrity, while ANGP-2 promotes permeability and remodeling under hypoxic or inflammatory conditions. In COPD, ANGP-2 expression correlates with vascular remodeling and disease severity [19,20,21], and circulating ANGP-2 predicts poorer outcomes across PH phenotypes [22,23,24]. Data on ANGP-1 are less consistent, though some evidence suggests involvement in vascular pathology [24].

In the absence of definitive circulating biomarkers reflecting pulmonary vascular involvement in COPD, this study investigated serum ANGP-1 and ANGP-2 as potential non-invasive indicators of echo-estimated pulmonary-pressure burden. Their relationships with mPAP, RAP, and spirometric indices were evaluated, while recognizing that echocardiographic pressure categories are not equivalent to RHC-confirmed PH.

## 2. Methods

### 2.1. Study Design and Ethics

This prospective, cross-sectional study was conducted at a tertiary care center in India. The protocol was approved by the institutional review board. Written informed consent was obtained from all participants. The study adhered to the International Council for Harmonization Good Clinical Practice Guidelines (2016), the Indian Council of Medical Research Ethical Guidelines (2017), and the Declaration of Helsinki (2013).

### 2.2. Participants

A two-sided comparison designed to detect a standardized mean difference of 0.70 at alpha = 0.05 and 80% power required approximately 33 participants per group [25]. Seventy consecutive participants with COPD were enrolled and subsequently stratified by the study-specific echocardiographic classification into an elevated e-mPAP COPD PH group (e-mPAP > 20 mmHg; *n* = 46) and a COPD without PH group (<20 mmHg; *n* = 24). Twenty additional control participants without a known diagnosis of COPD or PH were recruited and assessed using the same clinical, spirometric, echocardiographic, and biomarker procedures. COPD was diagnosed according to GOLD criteria (post-bronchodilator FEV1/FVC < 0.70) [1]. The e-mPAP threshold was used solely for non-invasive test echocardiography and was stratified into COPD PH and COPD without PH groups. Exclusion criteria included an acute exacerbation within 4 weeks, PH of another suspected etiology, clinically significant left-sided valvular disease or other structural left-heart disease, active infection, and malignancy. Age- and sex-matching of controls was attempted despite unequal group sizes.

### 2.3. Clinical and Functional Assessment

Smoking status, biomass exposure, comorbidities, exacerbation frequency, body mass index, and GOLD stage were recorded. Spirometry followed ATS/ERS standards [26].

### 2.4. Echocardiography

TTE was performed by a cardiologist. Peak tricuspid regurgitation velocity was measured using continuous-wave Doppler, and right ventricular systolic pressure was estimated as 4 × (peak tricuspid regurgitation velocity)^2^ + RAP. In the absence of right ventricular outflow tract or pulmonic valve obstruction, right ventricular systolic pressure was considered equivalent to pulmonary artery systolic pressure. RAP was estimated from inferior vena cava diameter and inspiratory collapse. Echo-estimated mPAP was then derived as 0.61 × pulmonary artery systolic pressure + 2 mmHg [27]. Comprehensive two-dimensional, color, and spectral Doppler assessment was used to identify clinically significant mitral or aortic stenosis or regurgitation, left ventricular systolic dysfunction, and other structural left-heart disease capable of increasing pulmonary pressure; affected patients were excluded. RAP, tricuspid annular plane systolic excursion, and left ventricular ejection fraction were also recorded.

### 2.5. Biomarker Analysis

Fasting venous blood samples were collected, centrifuged, and stored at −80 °C. Serum ANGP-1 and ANGP-2 were measured using ELISA (R&D Systems, Minneapolis, MN, USA). Detection limits were 50 pg/mL for ANGP-1 and 20 pg/mL for ANGP-2; intra-assay coefficients of variation were <5%.

### 2.6. Statistical Analysis

Data were analyzed using SPSS version 28. Distributional form was assessed using the Shapiro–Wilk test and graphical inspection. Approximately normally distributed variables were summarized as mean ± SD, whereas skewed variables were presented as median with interquartile range (IQR). Three-group comparisons used a one-way ANOVA or the Kruskal–Wallis test, as appropriate; pairwise nonparametric comparisons were performed using Mann–Whitney U tests with Holm adjustment. Categorical variables were assessed using chi-square or Fisher’s exact tests. Association analyses were restricted to the 70 COPD participants, with controls used for between-group biomarker comparisons. Pearson correlation coefficients were used for continuous variables and point-biserial coefficients for smoking status; 95% confidence intervals and analysis-specific sample sizes were reported, with Holm adjustment within each biomarker family. Multivariable linear regression models used ANGP-1 or ANGP-2 as the dependent variable and e-mPAP, RAP, and pre-bronchodilator FEV1 as predictors. Results are reported as unstandardized regression coefficients (B) with 95% confidence intervals. Model fit and assumptions were assessed using R^2^, adjusted R^2^, residual plots, the Jarque–Bera and Breusch-Pagan tests, and variance inflation factors. A two-sided *p* < 0.05 was considered statistically significant.

## 3. Results

### 3.1. Baseline Characteristics

The 70 consecutively recruited COPD participants were stratified into the COPD PH (*n* = 46) and COPD Without PH (*n* = 24) groups, and 20 controls were recruited additionally (Table 1). The two COPD groups were comparable in age, sex distribution, current smoking, comorbidities, symptoms, signs, and biomass exposure.

### 3.2. Hemodynamic and Functional Parameters

Median e-mPAP was higher in the COPD PH group [25.50 (IQR 23.00–30.00) mmHg] than in the COPD without PH group [16.00 (13.65–16.00) mmHg] and controls [16.00 (14.00–16.00) mmHg; overall *p* < 0.001] (Table 2). RAP showed the same pattern [12.00 (10.00–15.00), 5.50 (5.00–6.25), and 5.50 (5.00–6.00) mmHg; overall *p* < 0.001], although RAP was available for 46, 12, and 10 participants, respectively. The overall difference in pre-bronchodilator FEV1/FVC was modest (*p* = 0.035), and no pairwise comparison remained significant after Holm adjustment. Pre-bronchodilator FEV1 did not differ among groups (*p* = 0.370).

### 3.3. Serum Angiopoietin Levels

Median ANGP-1 was higher in the COPD PH group [8780.06 (IQR 7955.29–9902.88) pg/mL] than in the lower e-mPAP group [3065.14 (1170.69–6200.41) pg/mL] and controls [2997.19 (1292.13–3278.18) pg/mL; overall *p* < 0.001]. ANGP-2 showed a similar pattern [6152.92 (5380.27–8652.50), 2669.77 (1802.18–4744.00), and 2405.54 (1779.73–3751.76) pg/mL; overall *p* < 0.001]. The COPD without PH and control groups did not differ significantly for either biomarker after Holm adjustment. Among the 46 participants with e-mPAP > 20 mmHg, 23 had e-mPAP 20–25 mmHg and 23 had e-mPAP > 25 mmHg. ANGP-1 [8826.31 (7276.98–10,054.47) vs. 8738.95 (8086.33–9435.25) pg/mL; *p* = 0.930] and ANGP-2 [6898.22 (5395.95–8655.28) vs. 5822.49 (5425.75–8496.82) pg/mL; *p* = 0.750] were similar between these subgroups.

### 3.4. Correlations and Multivariate Associations

Among the 70 COPD participants, ANGP-1 correlated positively with e-mPAP (r = 0.640, 95% CI 0.477 to 0.761; *n* = 70) and RAP (r = 0.542, 95% CI 0.330 to 0.702; *n* = 58). ANGP-2 also correlated with e-mPAP (r = 0.545, 95% CI 0.355 to 0.691; *n* = 70) and RAP (r = 0.521, 95% CI 0.304 to 0.687; *n* = 58); all four Holm-adjusted *p* values were <0.001. The inverse association between ANGP-2 and pre-bronchodilator FEV1/FVC did not remain significant after Holm adjustment (adjusted *p* = 0.092). Neither biomarker was associated with pre-bronchodilator FEV1 or current smoking (Table 3).

The complete-case multivariable models included 70 COPD participants. For ANGP-1, e-mPAP (B = 157.52 pg/mL per mmHg, 95% CI 58.55 to 256.49; *p* = 0.002) and RAP (B = 326.36 pg/mL per mmHg, 95% CI 128.60 to 524.11; *p* = 0.002) were independently associated with higher concentrations, whereas pre-bronchodilator FEV1 was not (*p* = 0.340). For ANGP-2, RAP remained independently associated with concentration (B = 243.76 pg/mL per mmHg, 95% CI 85.78 to 401.74; *p* = 0.003), while e-mPAP (*p* = 0.166) and FEV1 (*p* = 0.794) were not. The ANGP-1 model had R2 = 0.481 and adjusted R2 = 0.450; the ANGP-2 model had R2 = 0.334 and adjusted R2 = 0.293.

### 3.5. Relationship Between Pulmonary Pressure and Angiopoietins

**Figure 1:**
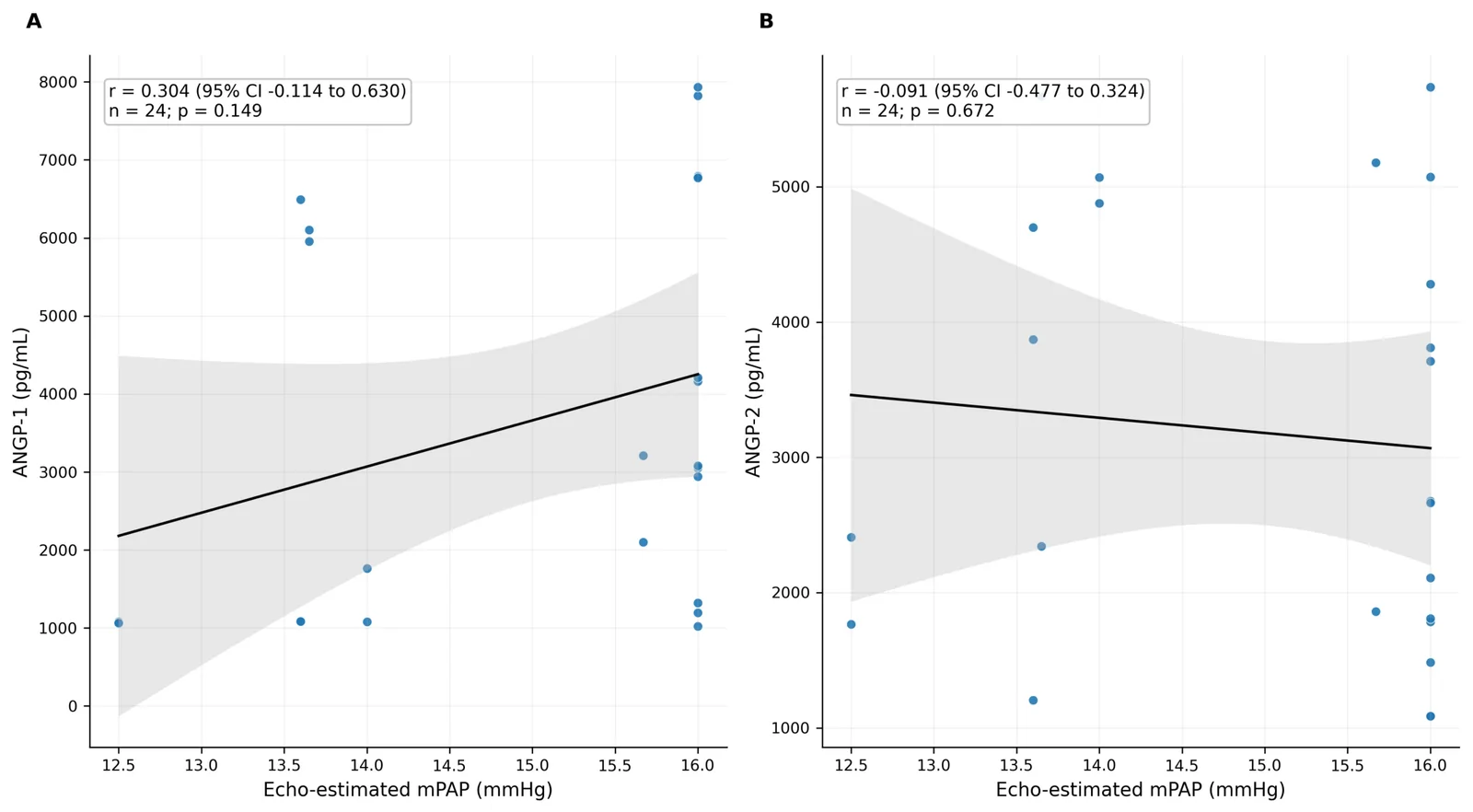
COPD participants with COPD without PH mPAP (<20 mmHg; *n* = 24). Separate relationships of (**A**) ANGP-1 and (**B**) ANGP-2 with e-mPAP. Lines show ordinary least-squares fits with 95% confidence bands.

Within the COPD without PH subgroup (*n* = 24), neither ANGP-1 nor ANGP-2 showed a statistically significant linear relationship with e-mPAP (ANGP-1: r = 0.304, 95% CI −0.114 to 0.630, *p* = 0.149; ANGP-2: r = −0.091, 95% CI −0.477 to 0.324, *p* = 0.672) (Figure 1). The wide confidence intervals indicate limited precision in this smaller subgroup.

**Figure 2:**
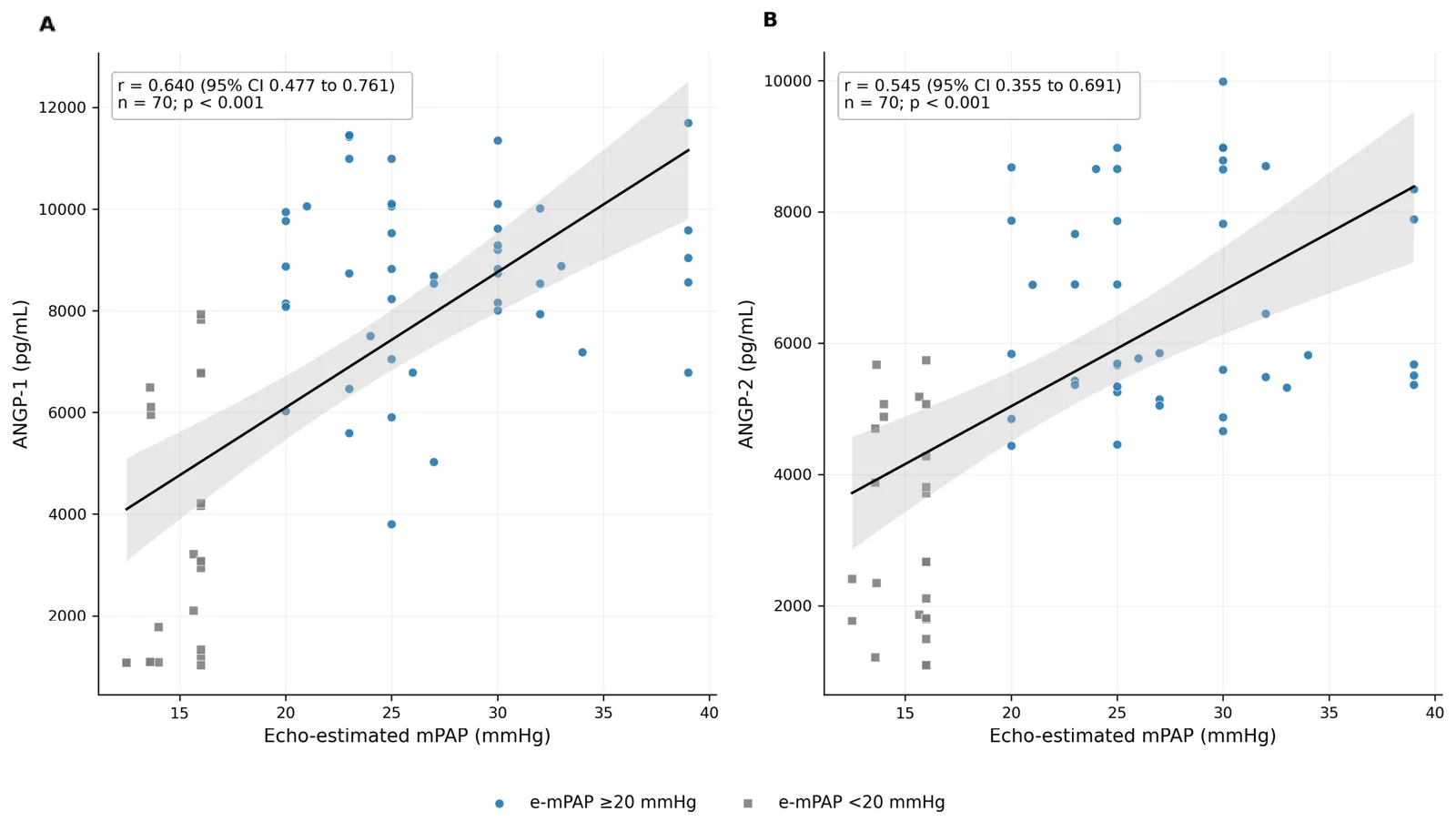
All COPD participants (*n* = 70). Separate relationships of (**A**) ANGP-1 and (**B**) ANGP-2 with COPD without PH (<20 mmHg) and COPD PH (≥20 mmHg) study groups were identified. Lines show ordinary least-squares fits with 95% confidence bands.

Across all 70 COPD participants, both biomarkers increased with e-mPAP (ANGP-1: r = 0.640, R2 = 0.410; ANGP-2: r = 0.545, R2 = 0.297; both *p* < 0.001) (Figure 2). However, biomarker concentrations were similar in the e-mPAP 20–25 mmHg and >25 mmHg subgroups, and correlations within the elevated e-mPAP group alone were not significant. The whole-cohort association should therefore be interpreted primarily as separation between lower and elevated echo-estimated pressure phenotypes rather than as evidence of a continuous dose–response relationship within established pressure elevation.

## 4. Discussion

In this study, circulating ANGP-1 and ANGP-2 concentrations were substantially higher in the COPD PH group than in those with COPD without PH and in controls. Within the COPD cohort, both biomarkers were associated with e-mPAP and RAP; after simultaneous adjustment for e-mPAP, RAP, and FEV1, e-mPAP and RAP remained associated with ANGP-1, whereas RAP remained associated with ANGP-2. These findings support an association with non-invasively estimated pulmonary artery pressure burden but do not establish a causal biomarker role.

The findings extend earlier work in COPD and PH while differing in important methodological respects. Garcia-Lucio et al. [19] reported an association between angiogenic-factor expression in pulmonary arteries and vascular remodeling in COPD, whereas Bessa et al. [21] described altered angiopoietin concentrations in induced sputum. Gouzi et al. [22] reported the circulating ANGP-2/ANGP-1 ratio as a marker of vascular impairment in COPD, and Cho et al. [23] observed increased ANGP-2 during acute COPD exacerbations. More recently, Enomoto et al. [24] reported diagnostic and prognostic associations of circulating ANGP-1 and ANGP-2 across PH phenotypes, while Ni et al. [20] found increased ANGP-2 in COPD-associated PH. The present study is consistent with higher circulating angiopoietins in an elevated pulmonary-pressure phenotype, but it specifically examined stable COPD using echocardiographic rather than invasive hemodynamic classification.

Experimental and translational studies provide biological plausibility for these associations. ANGP-1/Tie2 signaling has been linked to endothelial integrity and vascular adaptation [16,17,18,28,29,30], whereas ANGP-2 may antagonize Tie2 signaling in inflammatory or hypoxic conditions and increase vascular permeability [31,32,33]. Nevertheless, these mechanisms were not measured directly in the present cross-sectional cohort. The observed elevations should therefore be described as compatible with altered endothelial signaling rather than as proof that ANGP-1 represents a compensatory repair response or that ANGP-2 directly causes vascular destabilization.

The e-mPAP 20–25 mmHg subgroup is clinically relevant because it lies close to the contemporary hemodynamic threshold, yet echocardiography alone cannot confirm early PH. In this study, ANGP-1 and ANGP-2 were already elevated in this subgroup compared with the lower-pressure group, but their concentrations did not increase further in participants with e-mPAP >25 mmHg. This pattern suggests that angiopoietin elevation may accompany the transition to an elevated echo-estimated pressure phenotype, while the absence of a within-group gradient and the small subgroup size preclude conclusions regarding severity staging or therapeutic decision-making.

These results suggest that ANGP-1 and ANGP-2 may complement, but cannot replace, established clinical and echocardiographic assessment. Relative to the lower e-mPAP group, median ANGP-1 and ANGP-2 concentrations were approximately 186% and 130% higher, respectively; relative to controls, they were approximately 193% and 156% higher. These descriptive increases may inform future threshold-development studies, but a clinically applicable cut-off cannot be proposed from this cohort because no RHC reference standard, prespecified diagnostic-threshold analysis, or independent validation sample was available. The biomarkers should therefore not yet be incorporated into guideline-based Group 3 PH risk stratification.

Several limitations should be considered. The study was single-center, and the achieved lower e-mPAP COPD group (*n* = 24) was smaller than the approximately 33 participants per group indicated by the original power assumptions, limiting precision in subgroup analyses. Pulmonary pressure was estimated by TTE rather than measured by RHC; consequently, the groups represent study-specific echo-estimated pressure phenotypes and not hemodynamically confirmed PH categories [34]. The cross-sectional design and single biomarker measurement preclude causal or temporal inference. RAP and spirometric data were incomplete for some participants, reducing the multivariable complete-case sample to 53. Although multiplicity was addressed using Holm adjustment and regression diagnostics were satisfactory, residual confounding by comorbidities, medication use, or systemic inflammation remains possible. Finally, the study was not designed to derive or validate clinical angiopoietin cut-offs.

In conclusion, circulating ANGP-1 and ANGP-2 were higher in the COPD PH phenotype and were associated with e-mPAP and RAP within the COPD cohort. These observations are compatible with altered endothelial signaling in COPD-related pulmonary vascular involvement but do not establish a mechanism, diagnostic PH status, or a clinically actionable biomarker threshold. Larger longitudinal studies using RHC-based hemodynamic classification and independent validation cohorts are required.

## Figures and Tables

**Table 1:** Baseline characteristics across the three study groups.

Parameter	COPD PH (*n* = 46)	COPD Without PH (*n* = 24)	Controls (*n* = 20)
Age, mean ± SD (years)	55.15 ± 11.67	54.58 ± 12.55	52.95 ± 10.99
Sex, *n* (%)			
Male	32 (69.6)	17 (70.8)	15 (75.0)
Female	14 (30.4)	7 (29.2)	5 (25.0)
Current smoker, *n* (%)	20 (43.5)	8 (33.3)	7 (35.0)
Comorbidities, *n* (%)			
Diabetes mellitus	24 (52.2)	8 (33.3)	0
Hypertension	18 (39.1)	12 (50.0)	0
Symptoms, *n* (%)			
Dyspnea	26 (56.5)	15 (62.5)	0
Cough	21 (45.7)	10 (41.7)	0
Signs, *n* (%)			
Crepitations	24 (52.2)	14 (58.3)	0
History of biomass exposure, *n* (%)	22 (47.8)	9 (37.5)	0

None of the listed baseline characteristics differed significantly between the two COPD groups (all *p* > 0.05); the control group is shown descriptively.

**Table 2:** Comparison of biomarkers, echo-estimated hemodynamic measures, and spirometric parameters across groups.

Parameter	COPD with PH (*n* = 46)	COPD without PH (*n* = 24)	Controls (*n* = 20)	Overall *p* Value
ANGP-1 (pg/mL)	8780.06 (7955.29–9902.88)	3065.14 (1170.69–6200.41)	2997.19 (1292.13–3278.18)	<0.001
ANGP-2 (pg/mL)	6152.92 (5380.27–8652.50)	2669.77 (1802.18–4744.00)	2405.54 (1779.73–3751.76)	<0.001
e-mPAP (mmHg)	25.50 (23.00–30.00)	16.00 (13.65–16.00)	16.00 (14.00–16.00)	<0.001
RAP (mmHg)	12.00 (10.00–15.00)	5.50 (5.00–6.25)	5.50 (5.00–6.00)	<0.001
FEV1/FVC pre (%)	61.60 (59.23–63.46)	63.33 (61.60–68.00)	63.46 (61.90–68.00)	0.035
FEV1 pre (L)	0.96 (0.65–1.40)	1.23 (0.88–1.52)	1.11 (0.88–1.43)	0.370

Values are median (IQR) unless otherwise stated. Pairwise Holm-adjusted comparisons showed that the COPD PH group differed from both the COPD without PH group and controls for ANGP-1, ANGP-2, e-mPAP, and RAP (all adjusted *p* < 0.001), whereas the COPD without PH and control groups did not differ. RAP data were available for *n* = 46, *n* = 12, and *n* = 10; FEV1/FVC for *n* = 36, *n* = 24, and *n* = 20; and FEV1 for *n* = 41, *n* = 24, and *n* = 20. ANGP = angiopoietin; e-mPAP = echo-estimated mean pulmonary arterial pressure; RAP = right atrial pressure; FEV1 = forced expiratory volume in one second; FVC = forced vital capacity; IQR = interquartile range.

**Table 3:** Bivariate correlations and multivariable linear regression associations of ANGP-1 and ANGP-2 among COPD participants.

Parameter	ANGP-1 r (95% CI)	ANGP-2 r (95% CI)	ANGP-1 B (95% CI)	ANGP-2 B (95% CI)
e-mPAP	0.640 (0.477 to 0.761) *n* = 70; <0.001	0.545 (0.355 to 0.691) *n* = 70; <0.001	157.52 (58.55 to 256.49) *p* = 0.002	55.38 (−23.69 to 134.44) *p* = 0.166
RAP	0.542 (0.330 to 0.702) *n* = 58; <0.001	0.521 (0.304 to 0.687) *n* = 58; <0.001	326.36 (128.60 to 524.11) *p* = 0.002	243.76 (85.78 to 401.74) *p* = 0.003
FEV1/FVC (pre)	−0.054 (−0.304 to 0.202) *n* = 60; 1.000	−0.279 (−0.498 to −0.027) *n* = 60; 0.092		
FEV1 (pre)	−0.114 (−0.348 to 0.134) *n* = 65; 1.000	−0.143 (−0.374 to 0.104) *n* = 65; 0.508	623.86 (−678.10 to 1925.82) *p* = 0.340	−135.86 (−1175.97 to 904.26) *p* = 0.794
Current smoking	0.051 (−0.187 to 0.282) *n* = 70; 1.000	−0.004 (−0.238 to 0.232) *n* = 70; 0.977		

Correlation coefficients are Pearson r; the smoking coefficient is point-biserial r. Correlation *p* values were adjusted within each biomarker family using the Holm method. Regression coefficients are unstandardized B values. The multivariable models included e-mPAP, RAP, and pre-bronchodilator FEV1 and used complete cases (*n* = 53). Model diagnostics: ANGP-1 R2 = 0.481, adjusted R2 = 0.450, Breusch-Pagan *p* = 0.109, Jarque–Bera *p* = 0.664; ANGP-2 R2 = 0.334, adjusted R2 = 0.293, Breusch-Pagan *p* = 0.473, Jarque–Bera *p* = 0.317. Maximum variance inflation factor was 1.52. ANGP = angiopoietin; e-mPAP = echo-estimated mean pulmonary arterial pressure; RAP = right atrial pressure; FEV1 = forced expiratory volume in one second; FVC = forced vital capacity.

## Data Availability

All data generated or analyzed during this study are included in this article. Further inquiries can be directed to the corresponding author.

## Abbreviations

ANGP, angiopoietin; BNP, brain natriuretic peptide; COPD, chronic obstructive pulmonary disease; e-mPAP, echo-estimated mean pulmonary arterial pressure; ERS, European Respiratory Society; FEV1, forced expiratory volume in 1 s; FVC, forced vital capacity; IQR, interquartile range; mPAP, mean pulmonary arterial pressure; NT-proBNP, N-terminal pro-BNP; PH, pulmonary hypertension; RAP, right atrial pressure; RHC, right heart catheterization; TTE, transthoracic echocardiography.
